# Deltacoronavirus HKU11, HKU13, PDCoV (HKU15) and HKU17 spike pseudoviruses enter avian DF-1 cells via clathrin-mediated endocytosis in a Rab5-, Rab7- and pH-dependent manner

**DOI:** 10.1186/s13567-024-01442-3

**Published:** 2025-01-17

**Authors:** Qi-Zhang Liang, Chun-Miao Ji, Bin Wang, Wei Chen, Feng Cong, Yu Huang, Yao-Wei Huang

**Affiliations:** 1 Institute of Animal Husbandry and Veterinary Medicine, Fujian Academy of Agricultural Sciences, Fuzhou, China; 2 Guangdong Laboratory for Lingnan Modern Agriculture, College of Veterinary Medicine, South China Agricultural University, Guangzhou, China; 3 Guangdong Laboratory Animals Monitoring Institute, Guangzhou, China; 4 State Key Laboratory for Animal Disease Control and Prevention, South China Agricultural University, Guangzhou, China; 5 Department of Veterinary Medicine, Zhejiang University, Hangzhou, China

**Keywords:** Avian deltacoronavirus, porcine deltacoronavirus, entry pathway, pseudovirus, endocytosis

## Abstract

Porcine deltacoronavirus (PDCoV), also known as HKU15, is a swine enteropathogenic virus that is believed to have originated in birds. PDCoV belongs to the genus *Deltacoronavirus* (DCoV), the members of which have mostly been identified in diverse avian species. We recently reported that chicken or porcine aminopeptidase N (APN), the major cellular receptor for PDCoV, can mediate cellular entry via three pseudotyped retroviruses displaying spike proteins from three avian DCoVs (HKU11, HKU13, and HKU17). In the present work, to better understand how avian-origin CoVs may be transmitted to pigs, we investigated the unknown DCoV entry pathway in avian cells. We show that clathrin-mediated endocytosis is involved in the entry of these DCoV pseudoviruses into chicken-origin DF-1 cells. Pseudovirus entry was suppressed by means of pharmacological inhibitors, dominant-negative mutants, and siRNAs targeting various cellular proteins and signalling molecules, suggesting that PDCoV and avian DCoV pseudovirus entry into DF-1 cells depends on clathrin, dynamin-2, cathepsins and a low-pH environment but is independent of caveolae and macropinocytosis. Furthermore, we found that DCoV pseudovirus entry was linked to Rab5- and Rab7-dependent pathways. This is the first report demonstrating that these DCoVs utilize clathrin-mediated endocytosis pathways to enter avian-origin cells, providing new insights into interspecies transmission of DCoVs.

## Introduction

Coronaviruses (CoVs) exhibit a broad host range encompassing avian and mammalian species [1]. Within the genus *Deltacoronavirus* (DCoV), viruses have been identified predominantly in avian hosts [2–4], although porcine DCoV (PDCoV/HKU15) recently emerged as a globally disseminated swine enteropathogenic virus [5–7]. Notably, three instances of PDCoV infection in pediatric patients in Haiti have been reported, underscoring the potential public health implications of this viral pathogen [8]. Molecular clock analysis revealed a recent interspecies transmission event of PDCoV from birds to mammals [3, 9], revealing a close genetic relationship between PDCoV and sparrow CoV HKU17, with over 90% amino acid (aa) identity across their seven conserved replicase domains [3, 10]. Additionally, the PDCoV S protein shares 69.8% and 71.2% aa similarity and has a conserved structure with the corresponding domains in bulbul CoV HKU11 and munia CoV HKU13, respectively, suggesting a potential common evolutionary origin [2, 11]. In support of this deduction, we recently reported that aminopeptidase N (APN), one of the major receptors for PDCoV [12, 13], also mediates the cellular entry of pseudotyped retroviruses carrying the spike glycoprotein from avian DCoVs HKU11, HKU13 and HKU17, providing important evidence of transmission from wild birds to poultry and from birds to mammals [11].

Enveloped virus internalization is known to occur via two primary pathways; some viruses deliver their genomes to the cytosol by directly fusing with the plasma membrane, whereas others utilize the endocytic machinery of their host [14]. The endocytic pathways exploited by CoVs to gain entry into host cells include clathrin-mediated endocytosis (CME), caveolin/raft-mediated endocytosis (CavME), macropinocytosis, and non-clathrin/non-caveolin-mediated endocytosis, as well as lesser-known variations in these pathways. CME is the most highly conserved and well-understood endocytic pathway employed by viruses. Clathrin is assembled on the cytoplasmic face of the plasma membrane to form clathrin-coated pits (CCPs). The CCPs pinch off the cell membrane and mature into clathrin-coated vesicles (CCVs), which then transfer cargo into endosomes [15]. Severe acute respiratory syndrome (SARS)-CoV-2 [16], African swine fever virus (ASFV) [17], vesicular stomatitis virus (VSV) [18], fowl adenovirus serotype 4 [19] and hepatitis C virus (HCV) [20] enter certain cells via CME. Another major endocytotic pathway used by several enveloped viruses, including HCoV-229E [21], classical swine fever virus (CSFV) [22] and canine respiratory CoV (CRCoV) [23], uses the CavME for viral internalization. Ebola virus [24] and Nipah virus [25] enter cells via clathrin-independent macropinocytosis. In addition, some viruses can hijack multiple endocytic pathways to enter host cells; porcine epidemic diarrhea virus (PEDV) enters cells via CavME- and clathrin- and caveola-dependent pathways [26, 27], whereas transmissible gastroenteritis virus (TGEV) enters cells through clathrin- and caveolin-mediated endocytosis [28].

A previous study reported that PDCoV enters host cells via endocytic pathways and uses endosomal protease cathepsin L (CTSL) and cathepsin B (CTSB) for human cell entry, although proteases from the extracellular environment may also facilitate the cell entry process [29]. Later, Fang et al. reported that PDCoV enters porcine IPI-2I intestinal epithelial cells via macropinocytosis and clathrin-mediated endocytosis in a pH- and dynamin-dependent manner [30]. Li et al. demonstrated that PDCoV enters PK-15 cells through the CavME [31]. Interestingly, PDCoV does not require the participation of the endosomal system in ST cells [32]. However, the precise mechanisms for avian-origin cell entry by PDCoV and avian CoVs have never been studied, leaving a critical missing link in our understanding of bird-to-pig CoV transmission. Therefore, examining the cell entry of HKU11, HKU13, HKU17 and PDCoV in the same cell line will provide novel knowledge of the causes of PDCoV infections in pigs or other mammals.

In this study, we used avian-origin DF-1 cells as a cellular model to study the entry pathways of PDCoV and certain avian DCoVs, systematically perturbing the functions of key factors in various endocytic routes by using chemical inhibitors, siRNA silencing, and the overexpression of dominant negative (DN) mutant proteins. Our results indicate that CME is part of the DCoV infection process in DF-1 cells and that dynamin-2 and low pH are specifically needed. CTSL and CTSB inhibition decreased the entry of DCoV pseudovirions into DF-1 cells, and a reduction in Rab5 and Rab7 significantly inhibited entry by DCoV spike-pseudotyped retroviruses. Together, our data not only characterize avian DCoV entry and intracellular trafficking for the first time but also provide valuable information that can be used to evaluate the emerging disease potential of avian CoVs, enabling prevention or control of future outbreaks of DCoVs in mammals, including humans.

## Materials and methods

### Cells and plasmids

Chicken embryonic fibroblasts (DF-1; ATCC CRL-12203) and human embryonic kidney cells (HEK293T; ATCC CRL-11268) were individually grown in Dulbecco’s modified Eagle’s medium (DMEM) supplemented with 10% fetal bovine serum (Biological Industries) and 1% (w/v) penicillin and streptomycin (Gibco, USA). All the cells were grown at 37 °C with 5% CO_2_.

Dominant-negative (DN) mutants and constitutively active (CA) mutants of dynamin 2, Rab5 and Rab7 have been extensively reported in previous studies of enveloped viruses [33–35]. The genes DNM2 (Gene ID: 107051521), Rab5 (Gene ID: 420649), and Rab7 (Gene ID: 416016) were amplified via PCR from DF-1 cellular cDNAs and cloned and inserted into the vector pcDNA3.1 with a C-terminal Flag tag, generating Dynamin2-WT-Flag, Dynamin2-DN(K44A)-Flag, Rab5-WT-Flag, Rab5-DN(S34N)-Flag, Rab5-CA(Q79L)-Flag, Rab7-WT-Flag, Rab7-DN (T22N)-Flag, and Rab7-CA (Q67L). All primers used for plasmid construction are shown in Table 1.

**Table 1:** **Construction of mutant plasmids**

Primer	Sequence (5’–3’)
g-DNMII-arm-F	tccactagtccagtgtggtggaattcgccaccatggggaaccgcggcatggaggagctgatcccgctggt
g-DNMII-arm-R	tcagcgggtttaaacgggccctctagattacttatcgtcgtcatccttgtaatcgtcgagcagggagggct
g-DNMII-mut-F	tgccgcagatcgccgtggtgggcgggcagagcgcggggaagagctccgtgctggagaacttcgt
g-DNMII-mut-R	gaagttctccagcacggagctcttccccgcgctctgcccgcccaccacggcgatctgcggcaggt
g-Rab5-WT-F	atggctaatcgtggagcaacaagacccaacgggccaaat
g-Rab5-WT-R	cttatcgtcgtcatccttgtaatcgttactacaacatt
g-Rab7-WT-F	atgacttctaggaagaaagtgttact
g-Rab7-WT-R	cttatcgtcgtcatccttgtaatcgcagctgcagctct
g-Rab5-DN-F	agtctgcagttggtaaaaacagtttggtgct
g-Rab5-DN-R	agcaccaaactgtttttaccaactgcagact
g-Rab7-DN-F	gtggggaagaactcactcatgaaccagt
g-Rab7-DN-R	tcatgagtgagttcttccccaccccagagt
g-Rab5-CA-F	atacagctgggctagagcggtat
g-Rab5-CA-R	ataccgctctagcccagctgtat
g-Rab7-CA-F	atacagcaggcctagaacgattccagtct
g-Rab7-CA-R	agactggaatcgttctaggcctgctgtat

### Inhibitors and antibodies

Bafilomycin A1 (Baf-A1; Abcam, USA), NH_4_Cl (Sigma‒Aldrich, USA), amantadine (Abcam, USA), nystatin (Sigma‒Aldrich, USA), latrunculin B (Abcam, USA), dynasore (Merck, USA), E-64d (Sigma‒Aldrich, USA), the CTSL inhibitor Z-FY-CHO (Santa Cruz Biotechnology, USA), the CTSB inhibitor CA-074 Me (Santa Cruz Biotechnology, USA), the anti-dynamin-2 antibody (Ab) (Abcam, USA), the anti-Rab5 Ab (Beyotime Biotechnology, China), the anti-Rab7 Ab (Beyotime Biotechnology, China), the anti-HIV-1 p24 antibody (Abcam, USA), and the anti-CHC (clathrin heavy chain) monoclonal Ab (Thermo Fisher, USA) were used in this study.

The optimal concentrations of inhibitors for DF-1 cell viability were determined using a CCK-8 kit (Beyotime Biotechnology, China). DF-1 cells tolerated the following concentrations of these inhibitors at 95–100% viability: 2 mM for NH4Cl, 50 nM for Baf-A1, 50 μM for CPZ, 25 μM for nystatin, 100 μM for latrunculin B, 100 μM for dynasore, 50 μM for E64D, 50 μM for Z-FY-CHO, and 50 μM for CA-074. Therefore, we set these values as the maximum concentrations for each inhibitor and established the corresponding concentration gradients.

### Knockdown

The siRNAs were designed by RayBiotech (Guangzhou, China; Table 2). An siRNA with a control sequence unrelated to all known genes (siCtrl) was also designed and synthesized. DF-1 cells were transfected with the appropriate siRNA via the Lipofectamine RNAiMAX transfection agent (Invitrogen) according to the manufacturer’s instructions, and the knockdown efficiencies were quantified by western blotting [36]. Subsequent experiments were performed 24 h after transfection.

**Table 2:** **siRNA sequences**

Target gene	Sequence (5’–3’)
CHC (gallus)	CCGCCTACCTGTTGTTATT
Rab5 (gallus)	GCAGATGACAACAGTTTAT
Rab7 (gallus)	CCAGTATGTGAACAAGAAA
si-Nc (gallus)	TCAATCGGCTATGCATAAGT

### Pseudovirus cell entry assay

Retroviruses pseudotyped with S proteins from HKU11, HKU13, or HKU17 were packaged in 293T cells as described previously [11]. Briefly, pHIV-Luc (pNL4.3-HIV-Luc) and S expression plasmids (or empty vector as a control) were cotransfected into 293T cells using polyethylenimine (PEI). The produced pseudovirus particles were harvested at 48 h post-transfection. To determine the effects of the inhibitors on pseudoviral entry, DF-1 cells were infected with pseudoviruses pretreated with the inhibitors at 37 °C for 2 h. All the reporter assays were repeated at least three times.

### Western blot assays

For western blotting, the cells were lysed in 125 µL of CelLytic M lysis buffer (Sigma) per 10^6^ cells. The whole-cell lysates (WCLs) were used for SDS‒PAGE directly. The samples were resolved by SDS‒PAGE and transferred onto a polyvinylidene difluoride (PVDF) membrane that was subsequently blocked with Tris-buffered saline (TBS) containing 3% bovine serum albumin (BSA) overnight at 4 °C. Proteins were detected using a primary Ab followed by incubation with a horseradish peroxidase (HRP)-conjugated secondary Ab (Thermo Fisher Scientific).

### Statistical analyses

The data were analysed with GraphPad Prism 9 software, and two-tailed *t* tests were performed to determine significance. The data are expressed as the means ± standard errors of the means (SEM) of three independent experiments. *P* < 0.05 were considered statistically significant.

## Results

### HKU11, HKU13, HKU17 and PDCoV pseudovirus entry is dependent on low pH

**Figure 1:**
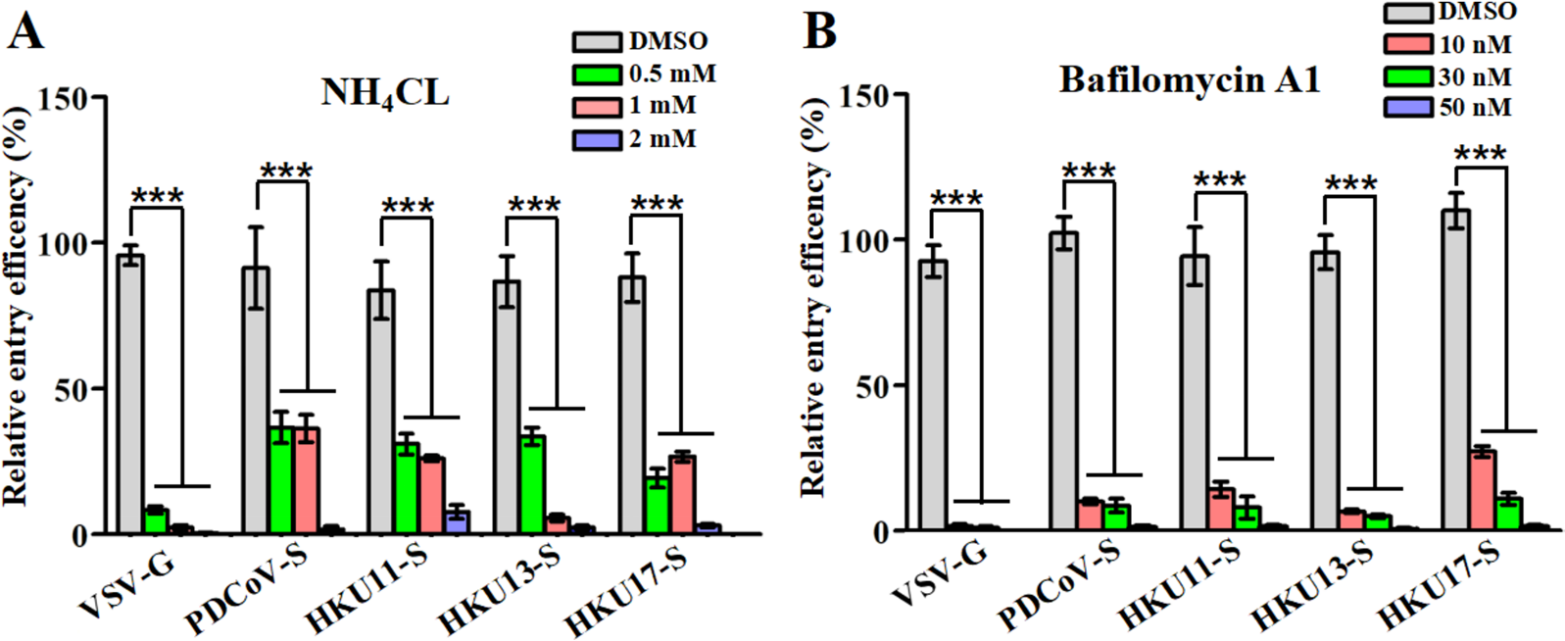
**HKU11, HKU13 and HKU17 pseudovirus entry requires an acidic endosomal pH.** DF-1 cells were pretreated with various concentrations of inhibitors at 37 °C for 2 h, and fresh DMEM with 10% FBS was added for 24 h. The optimal concentration of inhibitors for DF-1 cell viability was determined using a CCK-8 kit. DF-1 cells were preincubated with the endosomal acidification inhibitors **A** NH_4_Cl or **B** Baf-A1 at the indicated concentrations, after which the cells were infected with selected DCoV spike-pseudotyped retroviruses. VSV-G pseudovirions were used as a control. Luciferase activity was used as a measure of cell entry efficiency after 48 h; error bars indicate SEM (two-tailed *t* test, *p < 0.05, ***p < 0.001; *n* ≥ 3).

A number of viruses require exposure to an acidified environment after internalization via endocytosis for successful penetration and infection [37–39]. To determine the effects of pH on HKU11, HKU13, HKU17 and PDCoV pseudovirus infectivity, DF-1 cells were treated with the lysosomotropic agents NH_4_Cl and bafilomycin A1 (Baf-A1), and their effects on virus entry were evaluated. The subtoxic dose of these endosome acidification inhibitors was confirmed by a cell viability assay to be 2 mM NH_4_Cl or 50 nM Baf-A1. As the basic mechanism of pH-dependent endocytosis of VSV has been well documented, VSV-infected DF-1 cells were used as a positive control [40]. Consistent with previous reports, 2 mM NH_4_Cl or 50 nM Baf-A1 decreased the entry of VSV-G pseudovirions by more than 95% compared with that of untreated controls [40]. A greater than 90% reduction in transduction by DCoV pseudovirions was also shown when DF-1 cells were incubated with either NH_4_Cl or Baf-A1 (Figures 1A and B), indicating that the entry of HKU11, HKU13 and HKU17 pseudovirions into DF-1 cells is pH dependent.

### Clathrin-mediated endocytosis is involved in HKU11, HKU13, HKU17 and PDCoV pseudovirus entry

**Figure 2:**
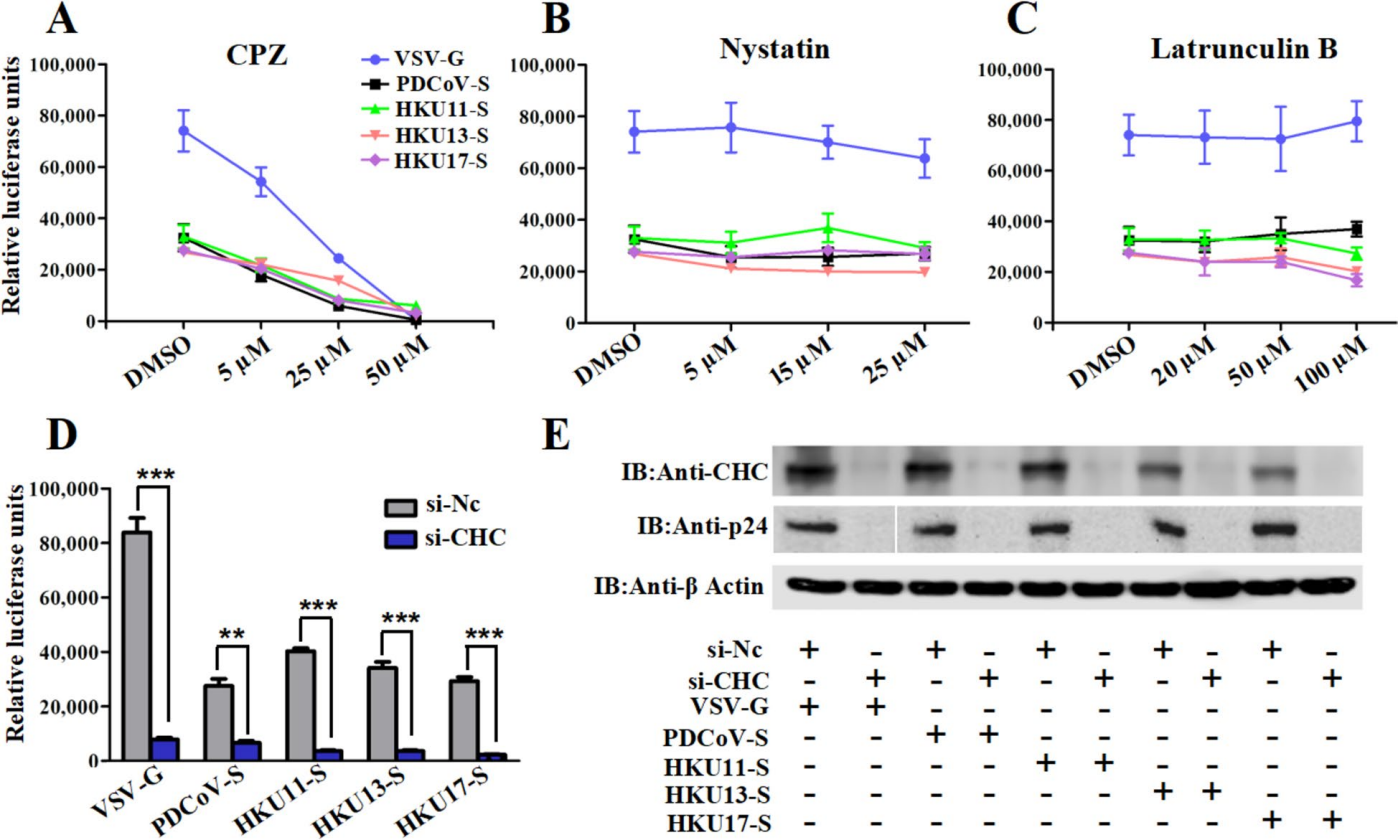
**CME pathways involved in HKU11, HKU13 and HKU17 pseudovirus entry into DF-1 cells.** DF-1 cells were pretreated with the indicated concentrations of **A** CPZ (CME inhibitor), **B** latrunculin B (macropinocytosis inhibitor), or **C** nystatin (CavME inhibitor) for 1 h and infected with retroviruses pseudotyped with selected DCoV spike proteins. The internalization of VSV-G pseudovirions via CME was used as a control. **D** siCHC- or siCtrl-transfected cells were infected with selected pseudoviruses. At 36 hpi, the cells were lysed to determine luciferase activity. **E** The expressed CHC or HIV p24 proteins were analysed by western blot; error bars indicate SEM (two-tailed *t* test, *p < 0.05, ***p < 0.001; *n* ≥ 3).

To better understand the pathway used by these DCoV pseudoviruses to enter DF-1 cells, we employed inhibitors of different endocytic mechanisms: chlorpromazine (CPZ), an inhibitor of clathrin-coated pit (CCP) formation [21]; nystatin, a well-known CavME inhibitor [30]; and latrunculin B, which inhibits macropinocytosis by disrupting actin polymerization [41]. As VSV entry is mediated by CME, the effectiveness of these inhibitors was first validated in VSV-infected cells. As expected, VSV-G entry was inhibited by CPZ but not by latrunculin B or nystatin (Figures 2A‒C). As shown in Figure 2A, CPZ, but not latrunculin B or nystatin, significantly inhibited avian DCoV pseudovirus entry, as evidenced by a dose-dependent reduction in luciferase activity.

Clathrin forms a triskelion shape composed of a clathrin heavy chain (CHC) and a light chain (CLC), and the former is known as a key component for the regulation of the formation and disassembly of the clathrin lattice [15]. To further evaluate the role of clathrin in avian DCoV pseudovirus internalization, CHC-specific siRNA was used to knockdown clathrin expression in DF-1 cells, after which the cells were infected with HKU11, HKU13 or HKU17 pseudovirions. A significant decrease in the luciferase activity of these avian DCoVs was observed in the siCHC-knockdown cells compared with those transfected with control siRNA (Figure 2D). The knockdown efficiency, reflected by the CHC expression level, and pseudovirus entry, indicated by the expression of the HIV capsid protein p24 [42], were validated by western blot analysis (Figure 2E). Taken together, these results strongly suggest that efficient HKU11, HKU13, HKU17 and PDCoV pseudovirus entry into DF-1 cells can occur via CME.

### HKU11, HKU13, HKU17 and PDCoV pseudovirus entry is sensitive to dynamin-2 inhibition

**Figure 3:**
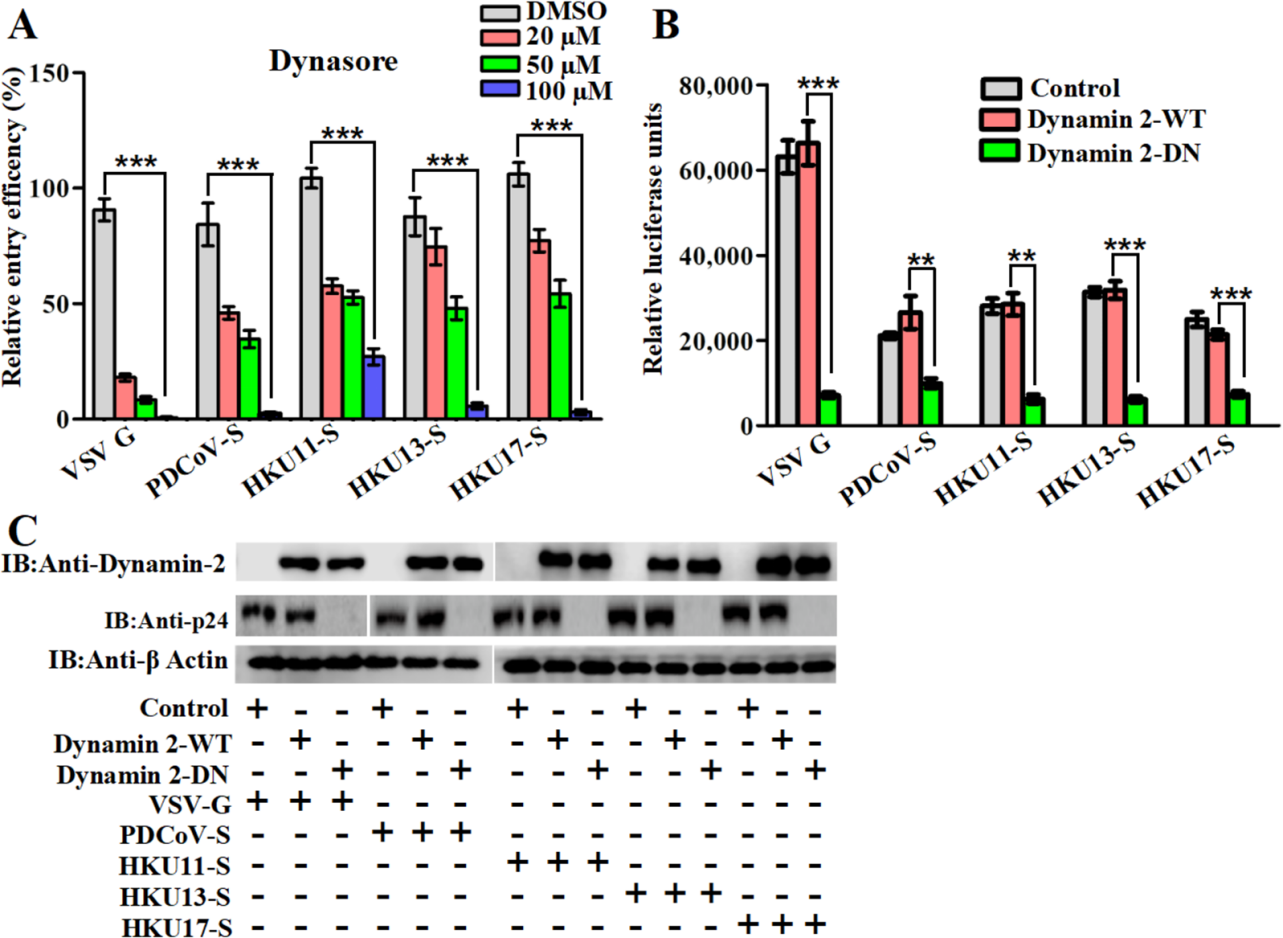
**HKU11, HKU13 and HKU17 pseudovirus entry depends on dynamin-2. A** DF-1 cells were pretreated with dynasore for 1 h at the indicated concentrations before pseudovirus infection, and the amount of virus endocytosed was measured by measuring luciferase activity at 48 hpi. **B** DF-1 cells were transfected with Flag-tagged WT dynamin-2 (DynII-WT) or DN mutant dynamin-2 (DynII-DN). At 24 h post-transfection, the cells were infected with selected pseudoviruses. The cell entry efficiency was measured by measuring the luciferase activity after 48 h. **C** The expression of these proteins as well as p24 was analysed via western blotting with an anti-Flag antibody; error bars indicate the SEM (two-tailed *t* test, *p < 0.05, ***p < 0.001; *n* ≥ 3).

Dynamin is a GTPase required for the cellular membrane to pinch off endosomes from the plasma membrane and is necessary for phagocytosis, CME and the CavME but is not required for macropinocytosis [43]. To determine whether ubiquitous dynamin-2 is involved in HKU11, HKU13, HKU17 and PDCoV pseudovirus entry, we first treated cells with the dynamin-2 inhibitor dynasore. As shown in Figure 3A, dynasore significantly inhibited pseudovirus entry, as evidenced by the notable reduction in luciferase activity with increasing dynasore concentrations up to 100 µM (Figure 3A).

The expression of a dominant-negative (DN) mutant of dynamin 2 (K44A) is known to prevent normal clathrin-mediated endocytosis [33]. Next, plasmids expressing wild-type (WT) and DN (K44A) dynamin-2 were transfected into DF-1 cells, which were subsequently infected with HKU11, HKU13, HKU17 and PDCoV pseudoviruses, with an empty pcDNA3.1 vector transfected as a control. DF-1 cells infected with VSV or PDCoV were used as positive controls. As shown in Figure 3B, compared with the WT controls, DN (K44A) expression significantly inhibited the entry of avian DCoV pseudoviruses into cells compared with that in the control group. Consistently, western blot analysis revealed that the expression of HIV p24 in pseudoviruses was significantly inhibited by the expression of DN (K44A) (Figure 3C). These results indicate that HKU11, HKU13, HKU17 and PDCoV pseudovirus entry into DF-1 cells is dependent on dynamin-2.

### Effects of cathepsin inhibitors on HKU11, HKU13, HKU17 and PDCoV pseudovirus entry

**Figure 4:**
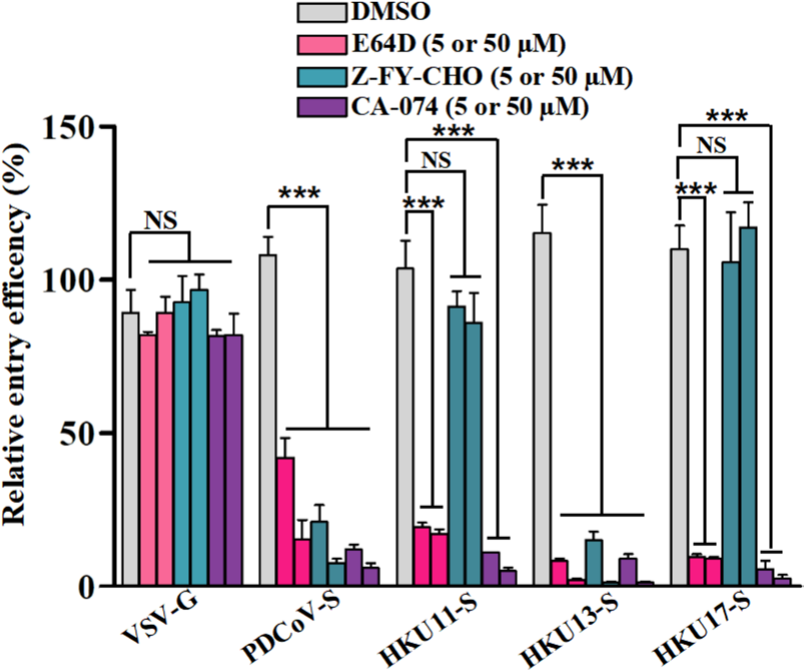
**Role of cathepsin proteases in HKU11, HKU13 and HKU17 pseudovirus entry into DF-1 cells**. DF-1 cells were pretreated with the broad-spectrum cathepsin inhibitor E64D, CTSL-specific inhibitor (Z-FY-CHO), CTSB-specific inhibitor (CA-074), or DMSO (as a negative control) and then transduced with selected pseudovirions. In the same drug treatment, there are two adjacent bars of the same color; the bar on the left is treated with 5 μM, and the bar on the right is treated with 50 μM. Pseudoviral transduction was measured by luciferase activity at 48 h post-inoculation, with VSV-G pseudovirus used as a negative control; error bars indicate SEM (*n* ≥ 3).

Concomitant with endocytosis, some viruses require CTSL- and/or CTSB-mediated cleavage of viral surface glycoproteins at various stages: before, during, or after fusion of the endosome with lysosomes to achieve infectivity [44]. To evaluate whether HKU11, HKU13, HKU17 and PDCoV pseudovirus entry is also dependent on cathepsins, we treated DF-1 cells with increasing concentrations of irreversible CTSL and the CTSB inhibitor E-64d, the CTSL inhibitor Z-FY-CHO, or the CTSB inhibitor CA-074. VSV-G pseudovirions were used as a negative control since virus entry mediated by VSV-G does not require protease activation. E64D treatment of DF-1 cells reduced the entry of avian DCoV pseudovirions by more than 90%, indicating that at least one cathepsin or calpain might be required for entry (Figure 4). While CTSL inhibitor treatment did not markedly affect HKU11 or HKU17 entry, CTSB inhibition decreased the entry of these avian DCoV pseudovirions into DF-1 cells by more than 90% at a CA-074 concentration of 50 µM (Figure 4), suggesting that cathepsin and CTSB, in particular, may be essential for priming HKU11, HKU13, HKU17 and PDCoV S proteins for entry into DF-1 cells.

### Rab5 and Rab7 are required for HKU11, HKU13, HKU17 and PDCoV pseudovirus entry

**Figure 5:**
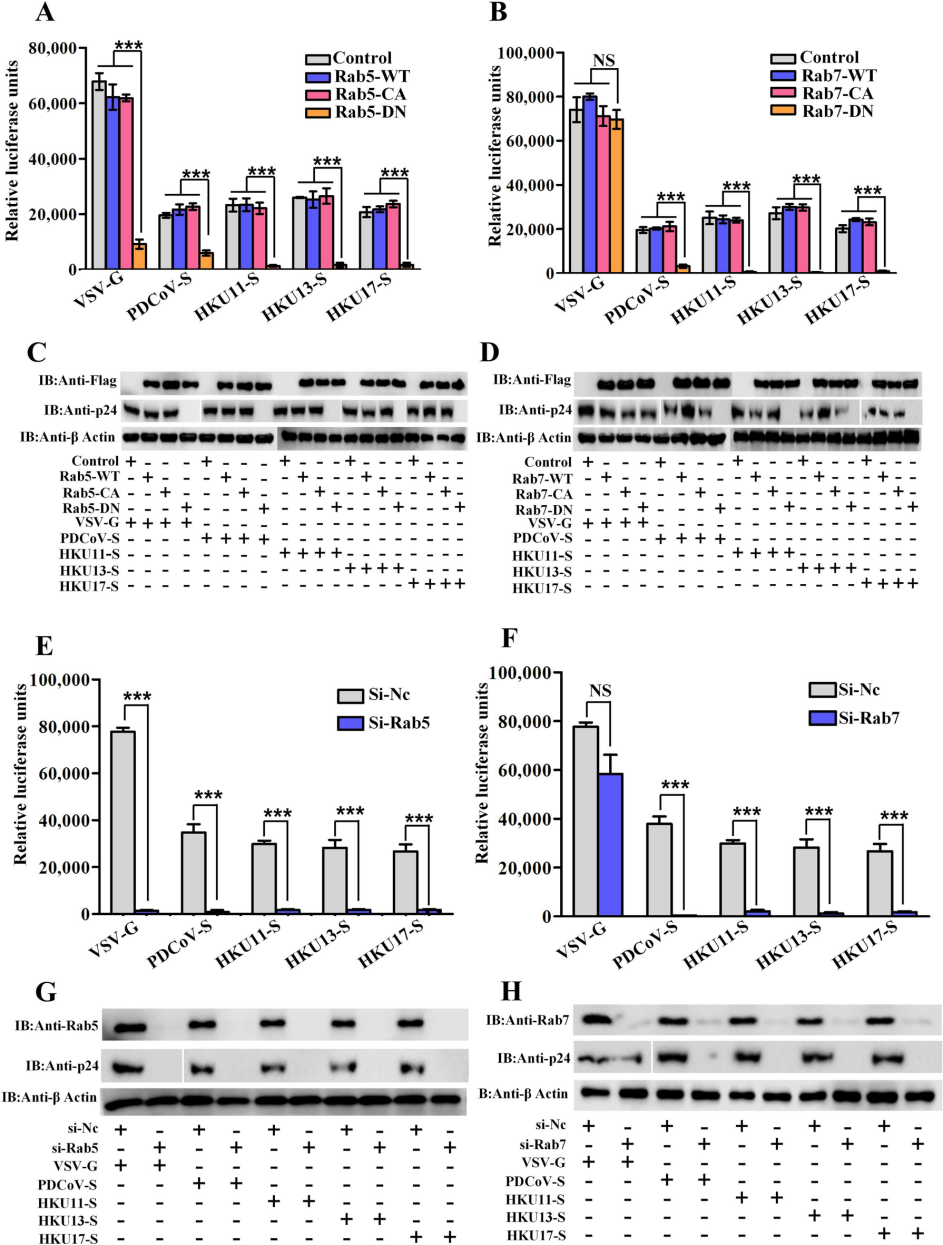
**Rab5 and Rab7 are necessary for HKU11, HKU13 and HKU17 pseudovirus infection. A**, **B** Cells transfected with plasmids expressing Flag-tagged Rab5 and Rab7 (WT or DN constructs) were then infected with select avian DCoV pseudoviruses. **C**, **D** The expression of these proteins as well as p24 was analysed via western blotting with anti-Flag antibodies. **E** siRab5- or **F** siRab7-transfected cells were infected with selected pseudoviruses, and the cell entry efficiency was determined by luciferase activity measurement after 36 hpi. **G**, **H** The knockdown efficiency of the siRNAs was measured by western blotting; error bars indicate SEM (two-tailed *t* test, *p < 0.05, ***p < 0.001; *n* ≥ 3).

Rab GTPases are a highly conserved family of proteins involved in early endosome formation and recycling, and Rab5 and Rab7 are known to be involved in vesicular trafficking to the early and late endosomal compartments, respectively (17, 22). We employed DN mutants of Rab5 (S34N) and Rab7 (T22N), which are known to block endocytosis at specific points, to probe the role of endosomal transport in the pseudoviral entry process [30]. In contrast, by overexpressing constitutively active (CA) mutants, the necessity of trafficking to early and late endosomes on avian DCoV pseudovirus entry could be confirmed [30, 34, 35]. DF-1 cells were first transfected with plasmids expressing the WT, DN (S34N) or CA (Q79L) forms of Rab5 as well as the WT, DN (T22N) or CA (Q67L) forms of Rab7. At 24 h post-transfection, the cells were infected with HKU11, HKU13, HKU17 and PDCoV pseudovirions. To ensure the functionality of the Rab constructs, we examined their effects on VSV infection. Consistent with previous reports, Rab5-DN, but not Rab7-DN, inhibited VSV infection (Figure 5A, B). HKU11, HKU13, HKU17 and PDCoV S protein pseudovirus entry was significantly inhibited in cells expressing DN Rab5/Rab7 but not in those expressing CA Rab5/Rab7 (Figures 5A, B). Accordingly, western blot analysis revealed that the expression of HIV p24 in pseudoviruses was significantly suppressed by DN Rab5/Rab7 (Figures 5C, D).

To confirm these findings, Rab5 or Rab7 was knocked down by transfection of DF-1 cells with siRab5 or siRab7, followed by pseudovirus infection. As shown in Figures 5G and H, compared with those in the siCtrl-transfected cells, both Rab5 and Rab7 expression were successfully knocked down. To validate the effects of Rab knockdown, we first examined the effect of Rab knockdown on VSV infection in DF-1 cells. As shown in Figures 5E and G, Rab5 knockdown reduced VSV-G pseudovirus entry, whereas Rab7 knockdown had no effect on VSV infection (Figures 5F and H). Measurement of luciferase activity revealed that knockdown of Rab5 or Rab7 significantly blocked HKU11, HKU13, HKU17 and PDCoV pseudovirus internalization (Figures 5E and F). Accordingly, the expression of HIV p24 in these pseudoviruses was significantly suppressed by siRab5 or siRab7 (Figure 5G, H). The above results demonstrate inefficient virus entry when the endosome transport pathways are disabled by the removal of Rab5 or Rab7.

## Discussion

The emergence of new and highly pathogenic CoVs, exemplified by SARS-CoV-2, has clearly outlined the threat posed by animal CoVs to human public health [45]. DCoVs have been identified in 30 families and across 108 species of wild birds [46]. Known avian DCoVs include seven species in three subgenera with extensive phylogenetic diversity and complex host ecology. Although first described in 2009 [2, 3], their primary receptors remained unknown until our recent report showing the importance of chicken or porcine APN as receptors for the avian DCoVs HKU11, HKU13 and HKU17, as in the case of PDCoV [11–13].

**Figure 6:**
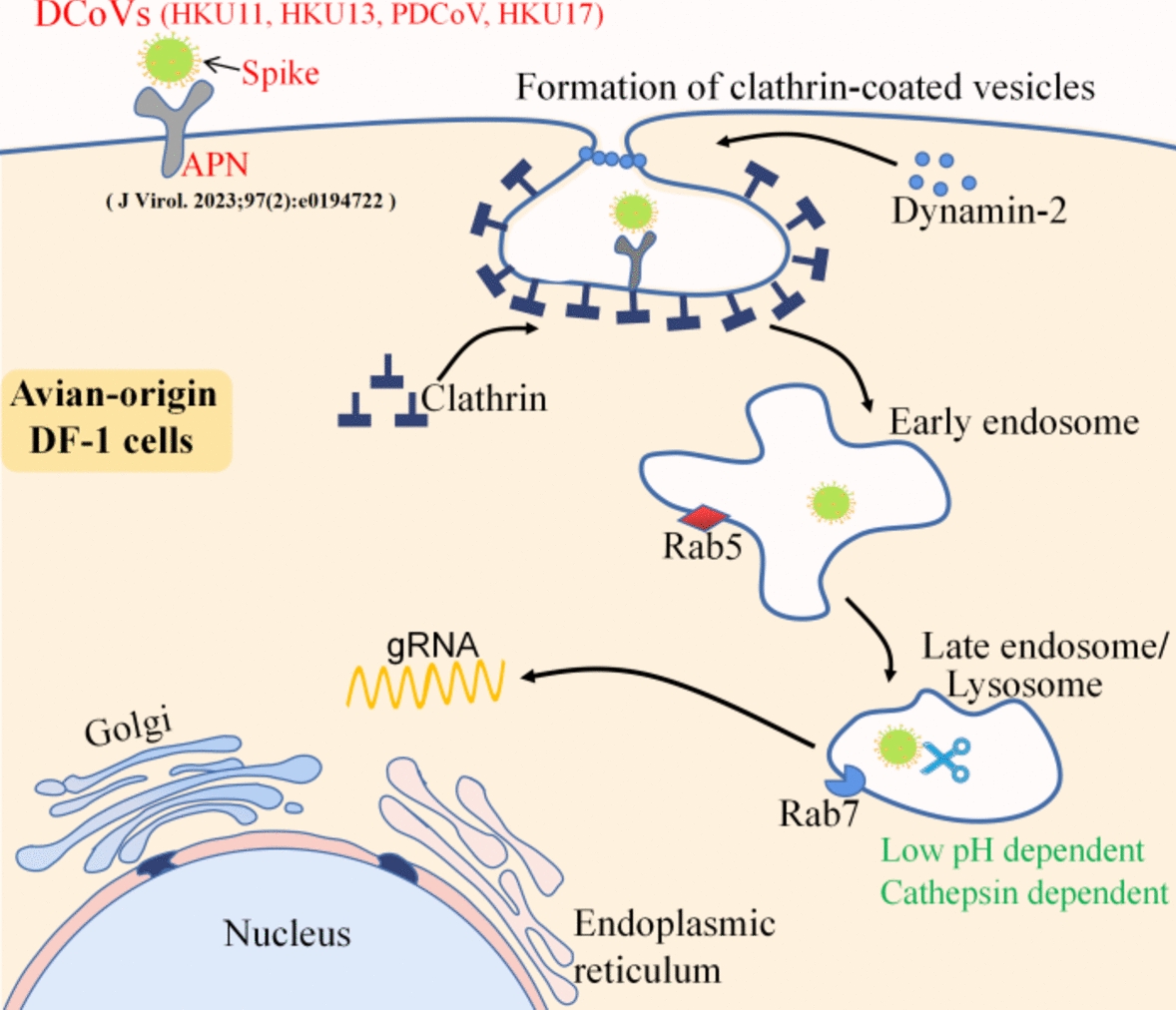
**Schematic illustrating HKU11, HKU13 and HKU17 pseudovirus entry into DF1 cells.** DCoV attaches to the cell surface and is internalized via clathrin-mediated endocytosis with the help of dynamin-2. The internalized virus is taken up into early endosomes in a Rab5a-dependent process. Through endosome maturation, virus-containing endosomes acquire Rab7 and become luminal components of Rab7-containing late endosomes. A low pH and cathepsin L/B are required for avian DCoV internalization and the subsequent transport steps to release viral genomic RNA (gRNA) into the cytoplasm.

As we tracked the first major part of the DCoV life cycle, the next question we addressed was the cellular receptor related to the entry pathway in avian cells. Most enveloped viruses are internalized via two primary pathways: some deliver their genomes to the cytosol by direct fusion with the plasma membrane, whereas others utilize the endocytic machinery of their host [14, 44]. Since no avian DCoVs have been isolated in cell culture thus far, the present study characterized the cellular entry of pseudotyped retroviruses displaying spike proteins from avian DCoVs HKU11, HKU13 and HKU17 as well as PDCoV in DF-1 cells. We found for the first time that these pseudoviruses utilize CME pathways to enter avian-origin DF-1 cells in a process dependent on dynamin-2, cathepsins, Rab5, Rab7 and a low-pH environment. A proposed model for this entry process is shown in Figure 6.

A previous study reported that PDCoV enters swine cells via multiple distinct endocytic pathways [29–32]. The present work is the first report of the PDCoV entry pathway in avian-origin DF-1 cells. Unlike entry into certain porcine cells, PDCoV entry into DF-1 cells is strongly dependent on CME, similar to the pathway used by DCoVs HKU11, HKU13 and HKU17. Rab GTPases are crucial regulators of endosomal transport and serve as key orchestrators of various membrane transport mechanisms in eukaryotic cells [47]. Specifically, Rab5 and Rab7 GTPases play essential roles in directing cargo to early and late endosomes, facilitating the cellular uptake of various substances [17]. Previous studies have shown that the transport of PDCoV into porcine-origin IPI-2I cells requires the involvement of the Rab5 and Rab7 GTPases [30]. The current investigation assessed the impact of Rab5 and Rab7 on DCoV entry into DF-1 cells by overexpressing DN mutants and knocking down the expression of Rab5 and Rab7 using siRNA. These findings suggest that Rab5 and Rab7 play a role in the entry of HKU11, HKU13, HKU17, and PDCoV into avian-origin DF-1 cells. The fact that all of these DCoVs also use APN as their primary cellular receptor may explain the similar cellular tropism that we reported in a previous study [11] and indicate a common molecular basis for interspecies transmission of DCoVs.

Notably, treatment with a CTSL-specific inhibitor did not affect the entry of HKU11 or HKU17, whereas PDCoV and HKU13 were both more sensitive. The roles of CTSL and CTSB in the entry of other viruses, such as SARS-CoV, MERS-CoV and SARS-CoV-2, have been extensively studied [48, 49]. Furthermore, PDCoV infection can increase CTSL and CTSB expression in vivo and in vitro, and the enzyme activity of CTSB increases following PDCoV infection [29]. Daniloski et al. conducted a genome-wide CRISPR screen to determine the host factors involved in SARS-CoV-2 infection, including CTSL, a gene with established roles in viral entry. Owing to its ubiquitous expression, CTSL is considered a promising drug target in the context of different viral and lysosome-related diseases. On the basis of the adaptability of HKU13 to CTSL, whether a similar scenario occurs in HKU13 and whether certain avian DCoVs could become the next transmission risk need to be further studied and evaluated.

## Data Availability

All data underlying the results are available as the article, and no additional source data are needed.
